# *Leptochloa chinensis* identified as a new reservoir host of southern rice black-streaked dwarf virus

**DOI:** 10.1007/s44297-026-00079-2

**Published:** 2026-06-25

**Authors:** Pengpeng Ren, Jianing Lei, Yuhua Qi, Mengnan Chen, Zhuangxin Ye, Zhiming Chen, Jianping Chen, Chuanxi Zhang, Junmin Li, Jiangxing Wu, Qianzhuo Mao

**Affiliations:** 1 State Key Laboratory for Quality and Safety of Agro-Products, Key Laboratory of Biotechnology in Plant Protection of MARA, Key Laboratory of Green Plant Protection of Zhejiang Province, Institute of Plant Virology, Ningbo University, Ningbo, China; 2 College of Plant Protection, Fujian Agriculture and Forestry University, Fuzhou, China; 3 Popularization of Agricultural Technical Station of Ningbo, Ningbo, China; 4 Integrated Technical Service Center of Rongcheng Customs, Fuzhou, China

**Keywords:** Southern rice black-streaked dwarf virus, *Leptochloa chinensis*, Reservoir host, *Sogatella furcifera*, Epidemiology

## Abstract

Southern rice black-streaked dwarf virus (SRBSDV) is a destructive pathogen of rice that is transmitted by the white-backed planthopper (WBPH, *Sogatella furcifera*). Identifying infectious reservoirs within the SRBSDV cycle is critical for developing effective disease management strategies. This research identifies Chinese sprangletop (*Leptochloa chinensis*), a noxious weed commonly found in rice ecosystems, as a previously unrecognized natural host of SRBSDV. SRBSDV infection was detected in *L. chinensis* samples collected from rice paddies exhibiting SRBSDV symptoms. Transcriptomic analyses, observation of SRBSDV virions, and typical profiles of SRBSDV-derived small interfering RNAs provided evidence of active, low-level, and asymptomatic viral infection. Genomic comparisons revealed minor genetic divergence in the viral RNA-dependent RNA polymerase (RdRP) gene, suggesting host-specific adaptation without compromising transmissibility. Further investigations using transmission experiments demonstrated that WBPHs microinjected with SRBSDV obtained from *L. chinensis* efficiently transmitted the virus to rice seedlings at a rate of 35.7%. This study emphasizes the necessity of integrating weed management into SRBSDV control strategies to disrupt viral reservoirs and mitigate outbreaks.

**Supplementary Information:**

The online version contains supplementary material available at 10.1007/s44297-026-00079-2.

## Introduction

Southern rice black-streaked dwarf virus (SRBSDV) is a devastating pathogen of rice (*Oryza sativa*) that was first identified in China in 2008 [1]. It belongs to the genus *Fijivirus* within the family *Spinareoviridae*. SRBSDV has a segmented genome consisting of ten double-stranded RNA segments (S1–S10), which encode both structural and non-structural proteins [2, 3]. In infected rice plants, SRBSDV causes significant physiological disruptions, including stunted growth, leaf curling, and the formation of white-to-dark brown waxy protrusions on stems. These symptoms ultimately lead to a substantial reduction in rice yields [1]. The virus is efficiently transmitted in a persistent manner by the white-backed planthopper (WBPH, *Sogatella furcifera*) [4, 5]. WBPH is critical for viral epidemics, and some biocontrol strategies targeting WBPH show partial efficacy [6, 7]. For effective control deployment, each stage of the disease cycle warrants critical attention.

Previous studies have identified several natural hosts of SRBSDV within the Poaceae family, including rice, maize (*Zea mays*), Chinese sorghum (*Coix lacryma-jobi*), *Avena fatua*, barnyard grass (*Echinochloa crusgalli*), *Eleusine indica*, and *Pennisetum flaccidum* [4, 8, 9]. In the meantime, the virus vector WBPH infests a variety of Poaceae plants, primarily feeding on rice and resorting to other hosts, such as maize and Chinese sorghum, during non-rice cropping seasons [4, 8]. Consequently, a wide range of Poaceae plants are presumed to serve as viral reservoirs, enabling transmission between cropping cycles and perpetuating epidemics in rice agroecosystems. The ability of SRBSDV to adapt to various Poaceae hosts, along with the feeding behavior of the WBPH, underscores its persistent threat to rice production.

Chinese sprangletop (*Leptochloa chinensis*), a C4 annual grass native to tropical Asia, ranks among the most destructive weeds in global rice ecosystems [10, 11]. Its ecological plasticity enables it to thrive in diverse environments, including dryland rice fields, while its capacity to behave as a perennial under favorable conditions has facilitated its invasion across Asia, Africa, and the Pacific [12, 13]. Despite its prevalence in rice paddies and shared habitat with WBPH, *L. chinensis* had not been investigated as a potential SRBSDV host prior to this study.

In August 2024, during a field survey of SRBSDV-affected rice paddies in Huzhou, Zhejiang Province, China, we observed *L. chinensis* plants proliferating abundantly within infected rice fields. Random sampling of these weeds and subsequent laboratory testing revealed SRBSDV positivity. Intriguingly, transmission experiments demonstrated that the virus in *L. chinensis* retained infectivity toward both WBPHs and rice seedlings, despite its limited replication efficiency in *L. chinensis*. These findings highlight the critical role of Poaceae weeds as cryptic reservoirs in SRBSDV epidemiology. A thorough understanding of the SRBSDV infection cycle is essential to develop integrated control frameworks that disrupt viral persistence [4, 6].

## Results

### Molecular identification of *L. chinensis* and detection of SRBSDV infection

**Fig. 1:**
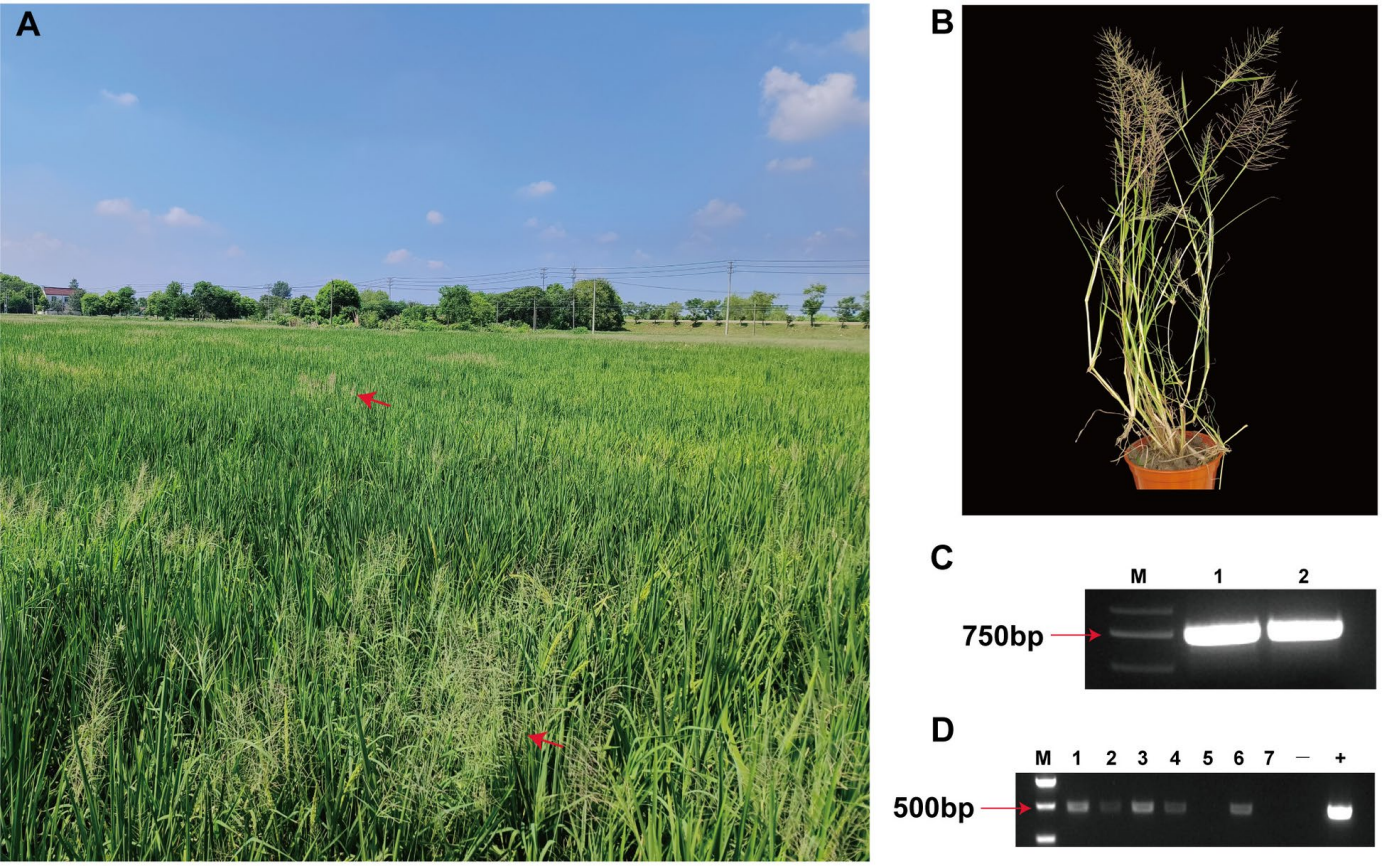
Field sampling, identification of *L. chinensis* and SRBSDV detection. **A** *L. chinensis* was prevalent in rice paddies in Huzhou, Zhejiang, China. **B** *L. chinensis* sampled randomly from SRBSDV-infested rice fields. **C** RT-PCR detection of internal transcribed spacer (ITS) from *L. chinensis*. The obtained ITS sequences demonstrated 100% identity with the *L. chinensis* reference sequence (GenBank accession number: KP057057). **D** Detection of SRBSDV in *L. chinensis* samples. Five out of seven *L. chinensis* samples tested positive for SRBSDV. *Abbreviations:* SRBSDV, southern rice black-streaked dwarf virus; RT-PCR, reverse transcription polymerase chain reaction

Seven grass weed samples suspected to be *L. chinensis* based on morphological characteristics were collected from SRBSDV-infected rice fields (Fig. 1A, B). Genomic DNA was extracted from samples and subjected to internal transcribed spacer (ITS) sequencing for species confirmation (Fig. 1C). BLAST analysis of the obtained ITS sequences demonstrated 100% identity with the *L. chinensis* reference sequence (GenBank accession number: KP057057), conclusively validating the taxonomic identity of the collected plants.

To assess SRBSDV infection, total RNA was extracted from all seven *L. chinensis plants* and subjected to RT-PCR targeting the S10 segment encoding the viral coat protein. SRBSDV-specific amplification was observed in five samples (71% infection rate, Fig. 1D), and the sequence was confirmed using Sanger sequencing, indicating natural viral presence in field-grown *L. chinensis*. The detection of SRBSDV in a majority of tested weeds, coupled with molecular validation of host identity, indicated that *L. chinensis* is a potential natural host.

### Low-titer replication of SRBSDV in *L. chinensis*

**Fig. 2:**
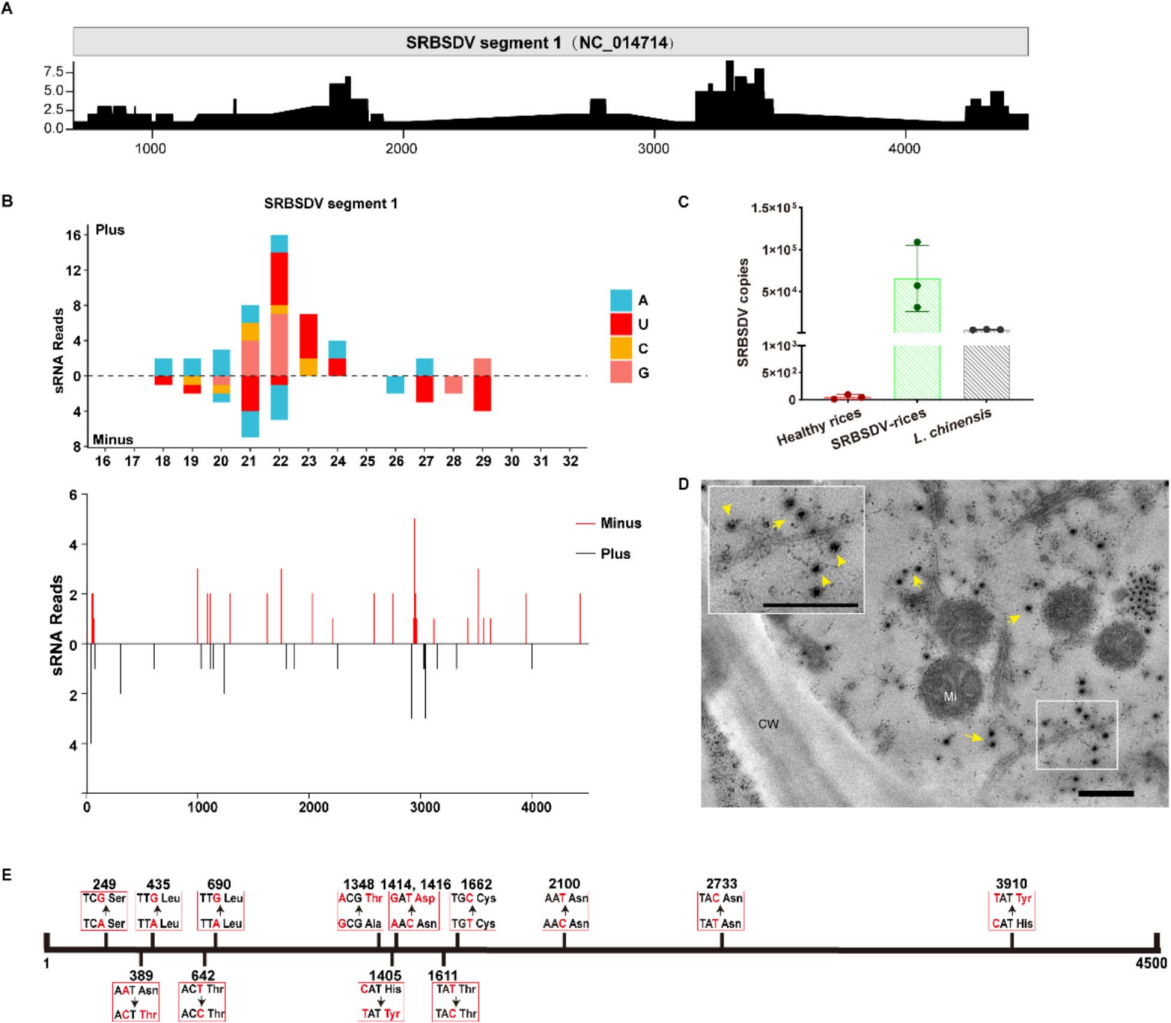
Analysis of SRBSDV infection and proliferation in *L. chinensis*. **A** Transcript coverage of SRBSDV S1. **B** Profile of virus-derived small interfering RNAs (vsiRNAs) of SRBSDV S1, peaking at 21 and 22 nt. **C** Detection of the proliferation levels of SRBSDV in rice and *L. chinensis*. Viral accumulation was significantly lower in *L. chinensis* than in infected rice plants (*p* < 0.01). **D** TEM observations of SRBSDV particles in *L. chinensis*. **E** Alignment of the SRBSDV S1 sequences from *L. chinensis* and rice. The 11 nucleotide differences and five nonsynonymous substitutions between *L. chinensis* and rice were consistently detected across all clones and replicates, confirming that they are genuine biological variations rather than PCR artifacts. *Abbreviations:* SRBSDV, southern rice black-streaked dwarf virus; TEM, transmission electron microscopy

To validate SRBSDV infection dynamics in *L. chinensis*, transcriptome sequencing and small RNA profiling were conducted on virus-positive samples. Transcriptome sequencing of SRBSDV-positive *L. chinensis* confirmed the presence of all ten viral genomic segments, albeit with low sequencing depth (average coverage < 10 ×), indicative of restricted viral transcription activity (Fig. 2A; Fig. S1). Small RNA sequencing identified SRBSDV-derived small interfering RNAs (vsiRNAs) predominantly 21–22 nt in length (Fig. 2B, Fig. S2), consistent with dicer-mediated processing patterns observed in rice, suggesting active viral replication and engagement of host RNA interference (RNAi) [14, 15]. Quantitative RT-PCR targeting the S10 segment (viral coat protein) revealed significantly lower viral accumulation in *L. chinensis* (1.14 × 10^4^ copies per μg total RNA) than in infected rice (1.34 × 10^5^ copies per μg total RNA) (*p* < 0.01; Fig. 2C). Transmission electron microscopy (TEM) further corroborated viral replication, revealing scattered double-layered spherical virions (~ 70 nm diameter) in phloem cells of asymptomatic leaves (Fig. 2E), which is morphologically characteristic of SRBSDV (Fig. 2D). Furthermore, full-length sequencing of the S1 segment (encoding RNA-dependent RNA polymerase, RdRP) from *L. chinensis* and rice samples collected in Huzhou fields. The SRBSDV S1 sequence from *L. chinensis* (GenBank accession number: PV636942) exhibited high similarity with both the NCBI reference (NC_014714; 99.78% nt, 99.80% aa) and co-localized field rice isolates (99.76% nt, 99.67% aa). Notably, 11 nucleotide differences in the SRBSDV S1 segment between *L. chinensis* and rice were identified, with five nonsynonymous substitutions resulting in amino acid alterations (Fig. 2E; Fig. S3; Fig. S4), highlighting minor genetic divergence. Collectively, these results demonstrate that SRBSDV establishes low-titer, asymptomatic infections in *L. chinensis*, supported by detectable vsiRNAs, virion visualization, and conserved viral genomes.

### SRBSDV transmission competences from *L. chinensis* to Rice via white-backed planthoppers

**Fig. 3:**
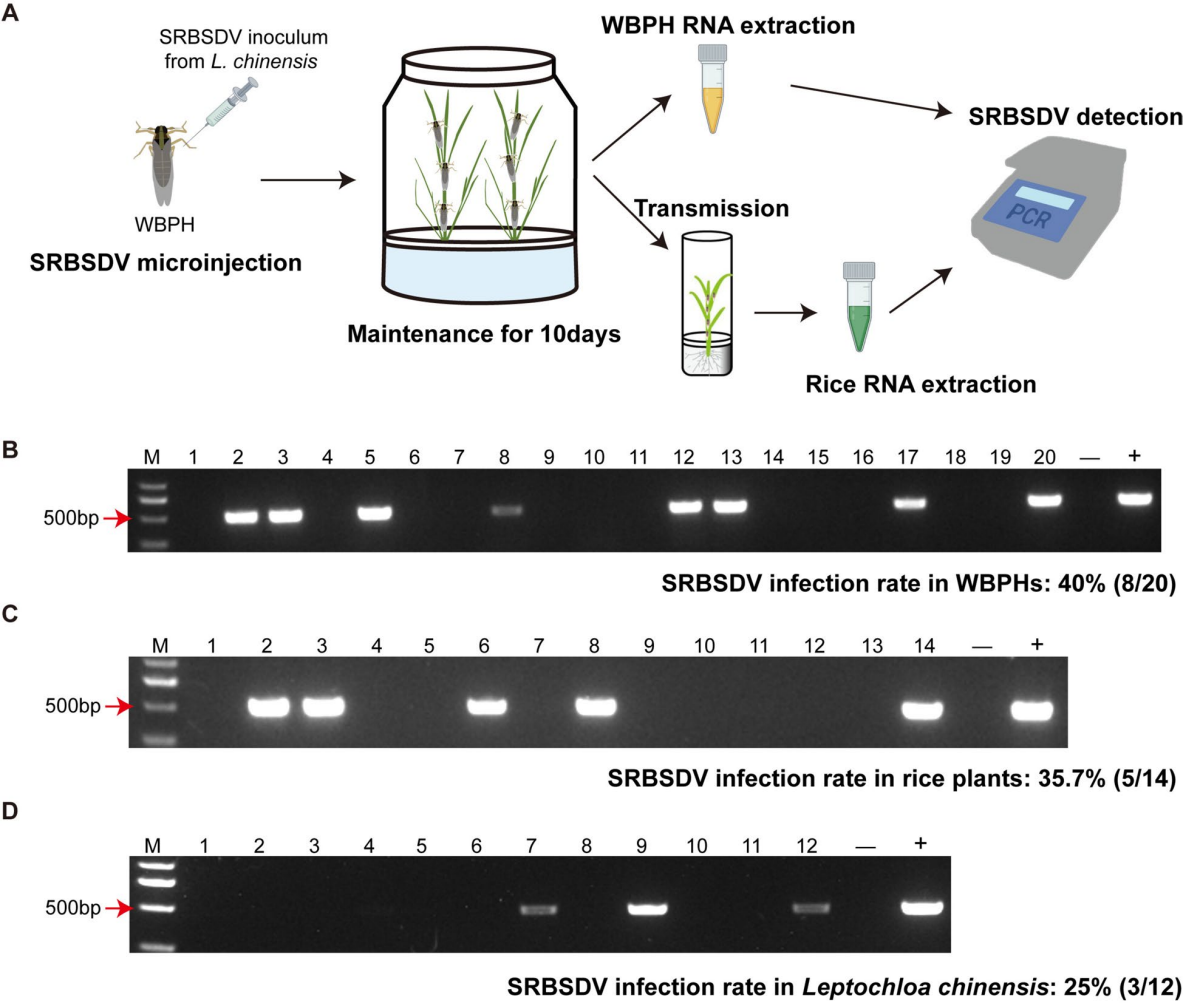
Assessing the infection capacity of SRBSDV from *L. chinensis*. **A** Overall strategy designed for the infection ability assessment of SRBSDV from *L. chinensis*. **B** RT‒PCR detection of SRBSDV in the WBPH after microinjection of SRBSDV from *L. chinensis* into the WBPH. At 8 days post-injection, 40% of WBPHs (8/20) tested positive for SRBSDV. **C** RT-PCR detection of SRBSDV in rice seedlings after feeding with microinjected WBPHs. SRBSDV infection was detected in 35.7% of exposed rice plants (5/14) at 15 days post-feeding. **D** RT-PCR detection of SRBSDV in *L. chinensis* seedlings after feeding with microinjected WBPHs. SRBSDV infection was detected in 25.0% of exposed rice plants (3/12) at 15 days post-feeding. *Abbreviations:* SRBSDV, southern rice black-streaked dwarf virus; RT-PCR, reverse transcription polymerase chain reaction; WBPH, white-backed planthopper

To assess the transmission potential of SRBSDV derived from *L. chinensis*, a crude viral extract prepared from infected weeds was microinjected into SRBSDV-free WBPHs (Fig. 3A). At 8 days post-injection, 40% of WBPHs (8/20) tested positive for SRBSDV via RT-PCR (Fig. 3B), confirming successful viral acquisition from the weed host. To evaluate transmission efficiency, injected WBPHs were allowed to feed on susceptible rice seedlings for 4 days. SRBSDV infection was detected in 35.7% of exposed rice plants (5/14) at 15 days post-feeding (Fig. 3C). These results demonstrate that SRBSDV extracted from *L. chinensis* retains infectivity in both its insect vector and primary crop host. Meanwhile, SRBSDV-infected WBPHS were fed healthy *L. chinensis* seedlings. At 15 days post-feeding, RT-PCR detection demonstrated that 25% (3/12) of the *L. chinensis* seedlings were infected with SRBSDV (Fig. 3D). These findings indicate that SRBSDV can be transmitted to *L. chinensis* via the feeding activity of WBPHs.

### Conclusions and discussion

This study presents the first evidence that *L. chinensis*, a pernicious Poaceae weed, serves as a natural host of SRBSDV. SRBSDV exhibits active, low-level, and asymptomatic infection in *L. chinensis*, as indicated by transcriptomic analyses, observation of SRBSDV virions, and typical profiles of SRBSDV-derived small interfering RNAs (Fig. 2). This finding has profound ecological and epidemiological implications.

As a pervasive noxious weed in rice agroecosystems, *L. chinensis* likely functions as a cryptic reservoir, facilitating viral transmission between cropping seasons. Its ecological adaptability and cohabitation with the WBPH vector amplify its potential role in sustaining SRBSDV epidemics. The high infection rate (71%) in field-collected *L. chinensis* specimens underscores its susceptibility to SRBSDV (Fig. 1D), even under low-titer conditions. This discovery expands the known host range of SRBSDV beyond cultivated rice. Although its quantitative role in regional virus persistence requires continuous monitoring, this weed likely provides refugia for viral survival during rice-free periods, potentially serving as a hidden source of inoculum [4, 8]. Current strategies focusing solely on vector control and symptomatic rice overlook alternative reservoirs such as *L. chinensis*. Integrated approaches, including weed eradication and disruption of vector-weed interactions, could effectively reduce viral carryover between seasons [7, 8, 16, 17].SRBSDV can infect a variety of Poaceae plants. In addition to rice and maize, the WBPH vector is able to acquire SRBSDV from weed hosts such as *Juncellus serotinus* and *Cyperus difformis* and then transmit the virus to other host plants [4, 8]. Transmission experiments using microinjection confirmed the infectivity of SRBSDV derived from *L. chinensis* into its vector, *S. furcifera* (Fig. 3). While microinjection bypasses the midgut barrier, it serves as a critical proof-of-concept that the virus harbored by this weed remains biologically potent. This confirms the weed's capacity to harbor infectious viruses, suggesting its potential role in sustaining SRBSDV transmission cycles. Although the viral titer in *L. chinensis* was observed to be lower than that in rice, several factors suggest that this weed is an ecologically relevant reservoir host intrinsically linked to SRBSDV epidemiology. First, the detection of vsiRNAs indicates active viral replication within the weed. Second, in field settings, the high density of *L. chinensis* around rice paddies may compensate for low individual transmission efficiency. Third, *L. chinensis* can be infected with SRBSDV through the feeding activity of WBPHs. Future studies utilizing natural feeding assays will be essential to quantify the precise acquisition efficiency and further elucidate the transmission dynamics between this weed host and rice crops. Comparative genomic analysis revealed minor genetic divergence in the RdRP gene between *L. chinensis*- and rice-derived isolates (Fig. 2E; Fig. S3), suggesting host-specific adaptation without compromising transmissibility. This plasticity underscores the evolutionary adaptability of SRBSDV across Poaceae hosts [18, 19].

In conclusion, this study identified the weed *L. chinensis* as a new reservoir host of SRBSDV. Its cryptic, low-titer infections may facilitate viral persistence and transmission, highlighting the potential role of such weeds in natural transmission cycles. Understanding these alternative hosts is essential for developing effective control strategies targeting both vectors and viral reservoirs to disrupt SRBSDV epidemics. Future research should explore the role of other Poaceae weeds, molecular drivers of viral adaptation, and the field-level efficacy of weed control in mitigating SRBSDV outbreaks. Such efforts are vital for safeguarding rice production against this evolving threat.

## Materials and methods

### Field sampling and morphological identification

Seven weed specimens were randomly collected in August 2024 from rice fields infected with SRBSDV in Huzhou, Zhejiang Province, China. Morphological analysis based on leaf structure, stem characteristics, and reproductive organs preliminarily identified the plants as Chinese sprangletop (*L. chinensis*). Samples were stored at − 80 °C for subsequent molecular analysis.

### Molecular species confirmation via ITS sequencing

Total genomic DNA was extracted from leaf tissues using the Wizard® Genomic DNA Purification Kit (Promega, USA) according to the manufacturer’s instructions. Species identity was validated by amplifying the internal transcribed spacer (ITS) region with primers ITS4 and ITS5 (Table S1) [20]. PCR reactions (50 µL total volume) comprised 1 µL template DNA, 5 µL 10 × PCR buffer, 5 µL dNTPs (2 mM), 2 µL MgSO₄ (25 mM), 1.5 µL each primer (10 µmol/L), 33 µL sterilized deionized water, and 1 µL KOD Plus Neo DNA polymerase (1.0 U/µL; Toyobo, Japan). Amplification was performed under the following conditions: initial denaturation at 94 °C for 2 min; 35 cycles of 98 °C for 15 s, 58 °C for 30 s, and 68 °C for 60 s; followed by a final extension at 68 °C for 10 min. PCR products were purified and sequenced commercially. Sequence homology was analyzed using the NCBI BLAST database (https://www.ncbi.nlm.nih.gov/) to confirm species identity.

### Virus detection and quantification

Total RNA was extracted from plant samples using the Trizol RNA Isolation Reagent (Takara, Japan) according to the manufacturer’s instructions. Reverse transcription polymerase chain reaction (RT-PCR) was employed to detect SRBSDV in the samples. For quantitative analysis, quantitative real-time PCR (qPCR) was performed on an ABI QuantStudio 5 system (Thermo Fisher Scientific, USA) as previously reported [21]. The 10-µL reaction mixture contained 2 µL of 20-fold diluted cDNA, 0.3 µL each of forward and reverse primers (10 µM), 5 µL Hieff qPCR SYBR Green Master Mix (Low Rox Plus) (Yiasen, Shanghai, China), and 2.4 µL nuclease-free water. The thermal cycling protocol included an initial denaturation at 95 °C for 5 min, followed by 40 cycles of 95 °C for 10 s (denaturation) and 60 °C for 30 s (annealing/extension). A melting curve analysis (95 °C for 15 s, 60 °C for 1 min, and 95 °C for 15 s) was conducted to confirm amplification specificity. A standard curve was generated using serial dilutions of a cloned SRBSDV S10 plasmid to quantify viral copy numbers, and the curve was y = −3.47x + 39.90 (R2 = 0.9817) (Fig. S5). The primers used in this study are listed in Table S1.

### Viral genome characterization

To confirm SRBSDV infection and compare viral genomes between *L. chinensis* and rice, cDNA synthesized from total RNA of both *L. chinensis* and rice served as the template for PCR amplification. Four primer pairs, designed based on the full-length SRBSDV S1 sequence (Table S1), were used to amplify overlapping fragments covering the RNA-dependent RNA polymerase (RdRP) region. PCR products were sequenced using Sanger and aligned with reference sequences using Clustal Omega. To ensure the accuracy of the S1 sequences, three independent SRBSDV-positive samples from both *L. chinensis* and rice were used as biological replicates. For each sample, the S1 segment was amplified, and at least three independent clones per PCR product were sequenced via Sanger sequencing to generate a consensus sequence and rule out potential PCR artifacts.

### Transcriptome and small RNA sequencing

For transcriptome analysis, high-quality RNA samples (OD260/280 = 1.8–2.2, RIN ≥ 8.0) from *L. chinensis* were sent to Novogene (Beijing, China) for library preparation and Illumina sequencing [22]. Poly(A) + RNA was isolated from 20 µg of total RNA using oligo(dT) magnetic beads, fragmented at 94 °C for 5 min in the presence of divalent cations, and reverse-transcribed into double-stranded cDNA (dscDNA) with N6 random primers. The dscDNA underwent end repair, adapter ligation, and PCR amplification, followed by purification with the QIAquick PCR Purification Kit (Qiagen, Hilden, Germany). Sequencing libraries were constructed and sequenced on an Illumina NovaSeq 6000 platform. The raw reads were first quality-trimmed to remove adapter sequences using Trinity software (Version 2.8.5) with default parameters and then subjected to de novo assembly of the clean reads [23].

For small RNA sequencing, libraries were prepared using the Illumina TruSeq Small RNA Library Prep Kit and sequenced on an Illumina HiSeq 2500 platform. The raw data were processed to remove adapters and low-quality sequences using the Cutadapt tool. Clean reads (16–32 nt) were mapped to the 10 SRBSDV reference genomes using Bowtie2 (v2.4.5) with strict parameters (no mismatches allowed). Viral genome coverage and small RNA profiles were analyzed using Samtools (v1.12) and custom Perl/Shell scripts [24, 25].

### Electron microscopy

The leaves were fixed overnight in 2% paraformaldehyde and 2.5% glutaraldehyde in PBS buffer, followed by an additional overnight fixation in 2% osmium tetroxide at 4 °C. After washing with PBS, the samples were dehydrated in a series of ethanol solutions (30%−100%) for 20 min each and then incubated in absolute acetone for 20 min. The samples were infiltrated with mixtures of Spurr resin and acetone (1:1 and 3:1) for 1 and 3 h, respectively, and embedded overnight in pure Spurr resin. The samples were polymerized in capsules at 70 °C overnight. Following staining with uranyl acetate and lead citrate for 5–10 min, the samples were examined using an HT7800 transmission electron microscope (Hitachi, Japan).

### Assessing the transmission capacity of SRBSDV from *L. chinensis*

WBPHs were reared in an artificial climate chamber at the Institute of Plant Viruses, Ningbo University, under controlled conditions: temperature 26 ± 0.5 °C, relative humidity 70% ± 5%, and a 16/8-h light/dark photoperiod. To evaluate the infectivity of SRBSDV derived from *L. chinensis*, viral crude extracts were prepared by homogenizing SRBSDV-infected *L. chinensis* leaves in 0.01 M phosphate-buffered saline (PBS, pH 7.4). Third-instar WBPH nymphs were microinjected with the crude extract using a FemtoJet microinjector (Eppendorf, Germany). Injected insects were transferred to rice seedlings (2–3 leaf stage) and maintained under the same climatic conditions. At 8 days post-injection (DPI), individual WBPHs were collected and subjected to SRBSDV detection using RT-PCR. Meanwhile, 30 microinjected WBPHs were transferred to healthy rice seedlings (2–3 leaf stage) for a 4-day feeding period. After removing the insects, rice plants were grown for an additional 15 days under controlled greenhouse conditions (28 °C, 70% humidity, natural light). Systemic leaves of rice seedlings were sampled, and SRBSDV was detected. In addition, 30 WBPHs infected by SRBSDV were fed healthy *L. chinensis* seedlings (2–3 leaf stage) for a 10-day period. After removing the insects, the *L. chinensis* plants were continuously grown for an additional 15 days under controlled greenhouse conditions (28 °C, 70% humidity, natural light). Subsequently, systemic leaves of the *L. chinensis* seedlings were sampled and subjected to SRBSDV detection.

## Supplementary Information

Supplementary Material 1: Fig. S1 Coverage of SRBSDV by transcripts from *L. chinensis*.

Supplementary Material 2: Fig. S2 Profile of virus-derived small interfering RNAs (vsiRNAs) of SRBSDV.

Supplementary Material 3: Fig. S3 Alignment of SRBSDV S1 nucleotide sequences from *L. chinensis* and rice.

Supplementary Material 4: Fig. S4 Alignment of SRBSDV S1 amino acid sequences from L. chinensis and rice.

Supplementary Material 5: Fig. S5 The standard curve for quantitative real-time PCR (qPCR).

Supplementary Material 6: Table S1 Primers used in this study.

## Data Availability

The authors declare that all data supporting the findings of this study are available in the manuscript, and its Supplementary Information files are available from the corresponding authors upon request.
