# Supplementary figures and images for: Leptochloa chinensis identified as a new reservoir host of southern rice black-streaked dwarf virus
Source: Crop Health. 2026 Jun 25;4(1):17. doi: 10.1007/s44297-026-00079-2 (PMC13304047; doi:10.1007/s44297-026-00079-2)

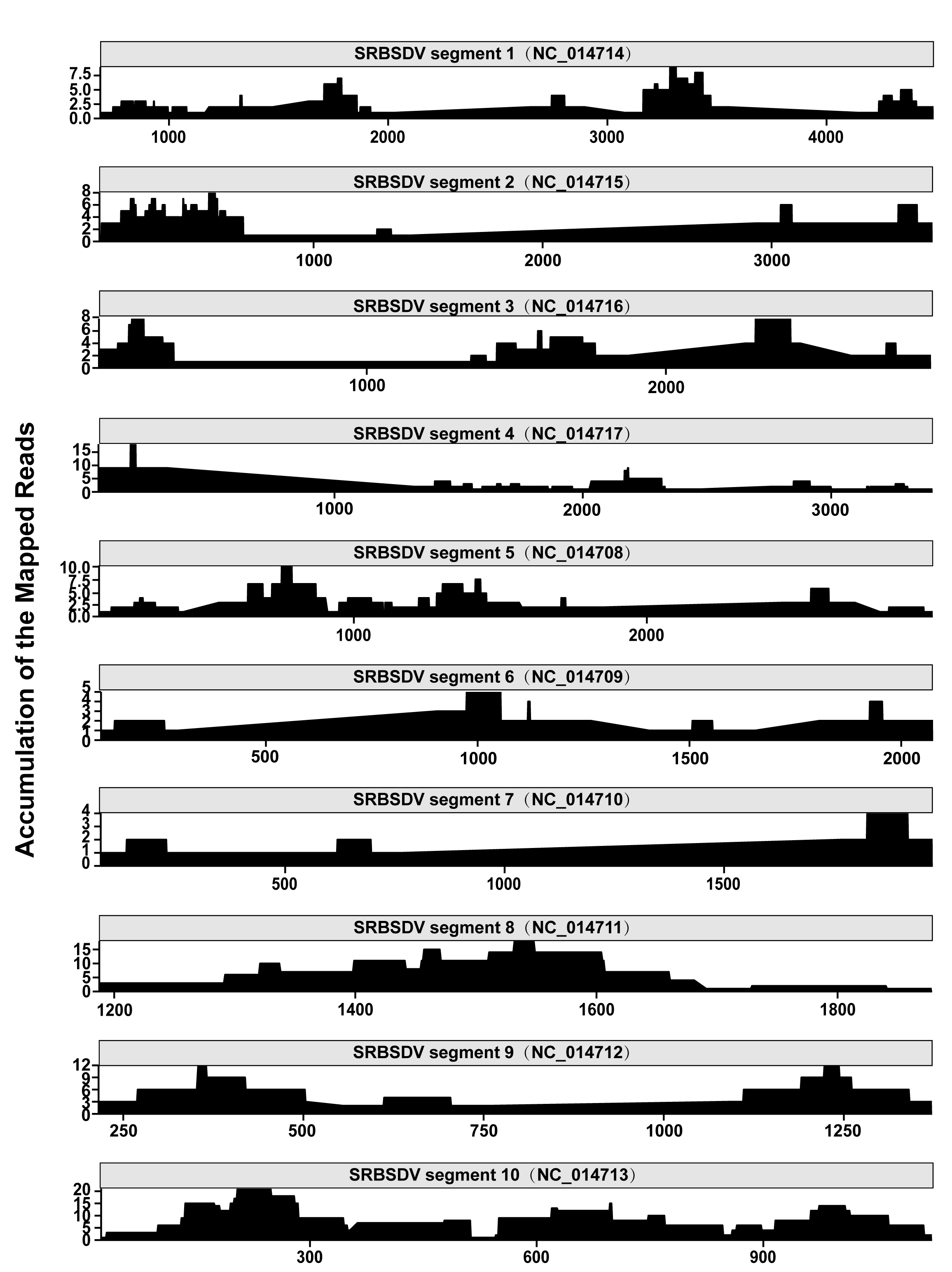

Supplement: Supplementary file 1 — Supplementary Material 1: Fig. S1 Coverage of SRBSDV by transcripts from L. chinensis. [file 44297_2026_79_MOESM1_ESM.tif]

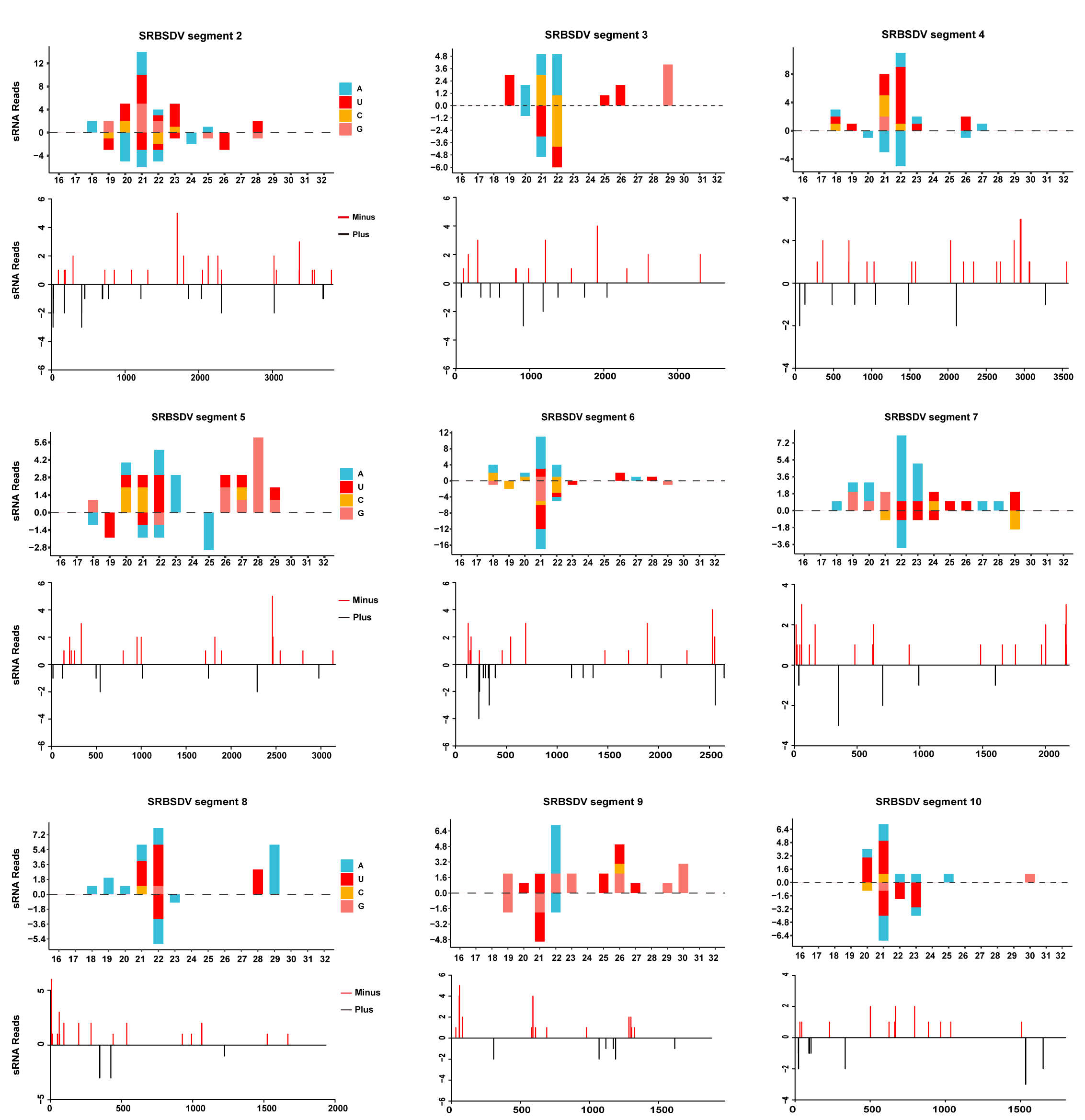

Supplement: Supplementary file 2 — Supplementary Material 2: Fig. S2 Profile of virus-derived small interfering RNAs (vsiRNAs) of SRBSDV. [file 44297_2026_79_MOESM2_ESM.tif]

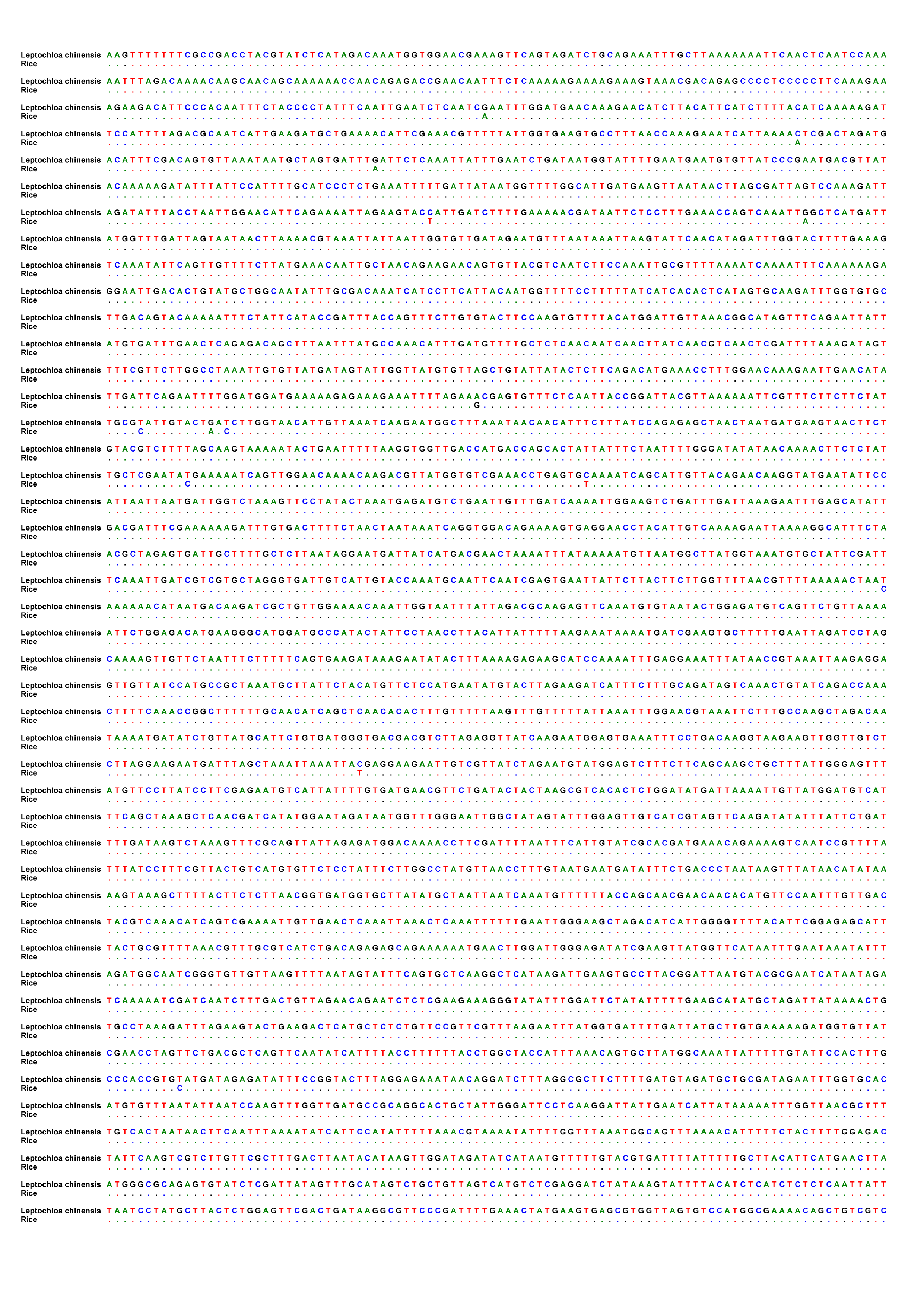

Supplement: Supplementary file 3 — Supplementary Material 3: Fig. S3 Alignment of SRBSDV S1 nucleotide sequences from L. chinensis and rice. [file 44297_2026_79_MOESM3_ESM.tif]

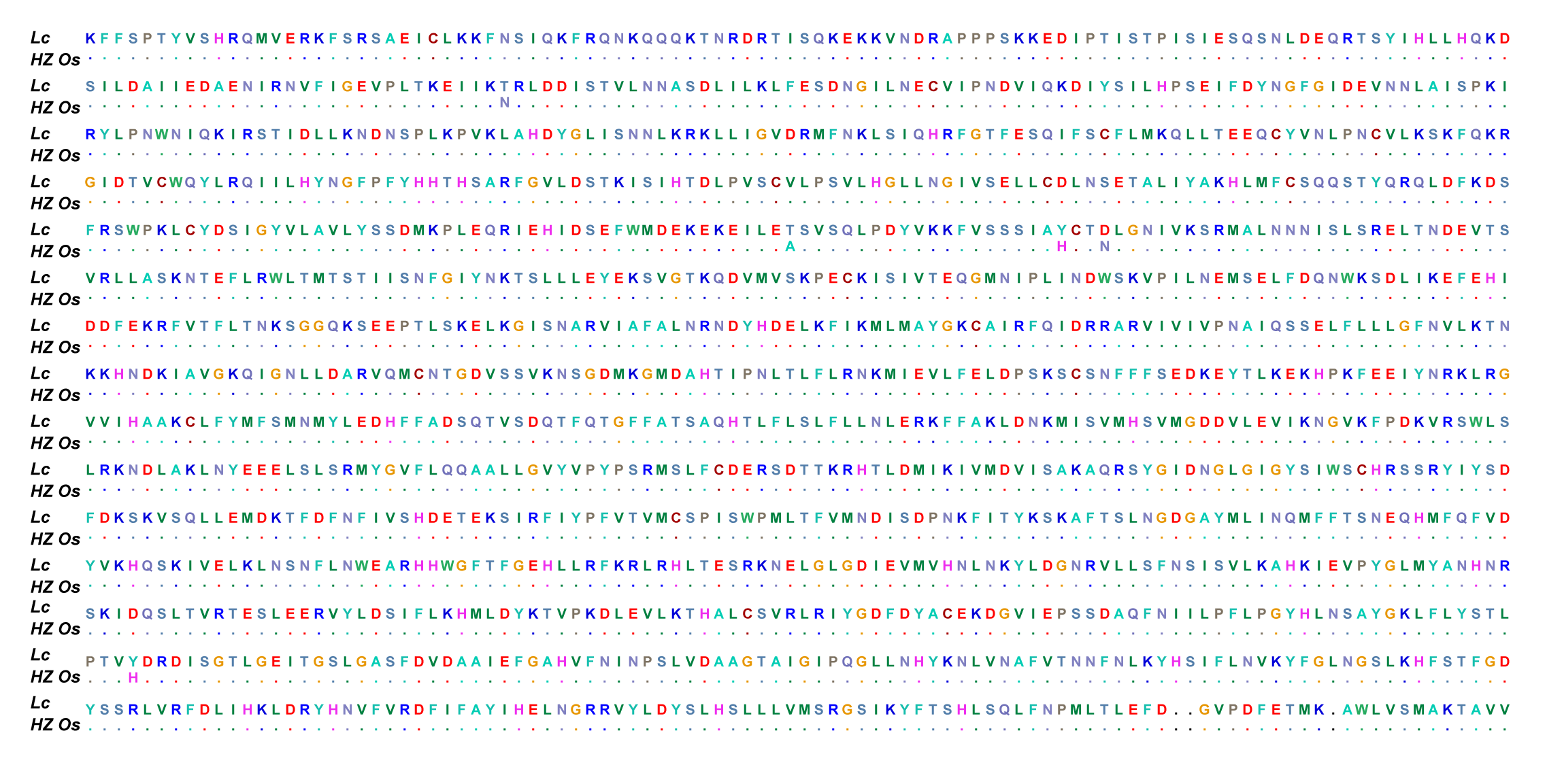

Supplement: Supplementary file 4 — Supplementary Material 4: Fig. S4 Alignment of SRBSDV S1 amino acid sequences from L. chinensis and rice. [file 44297_2026_79_MOESM4_ESM.tif]

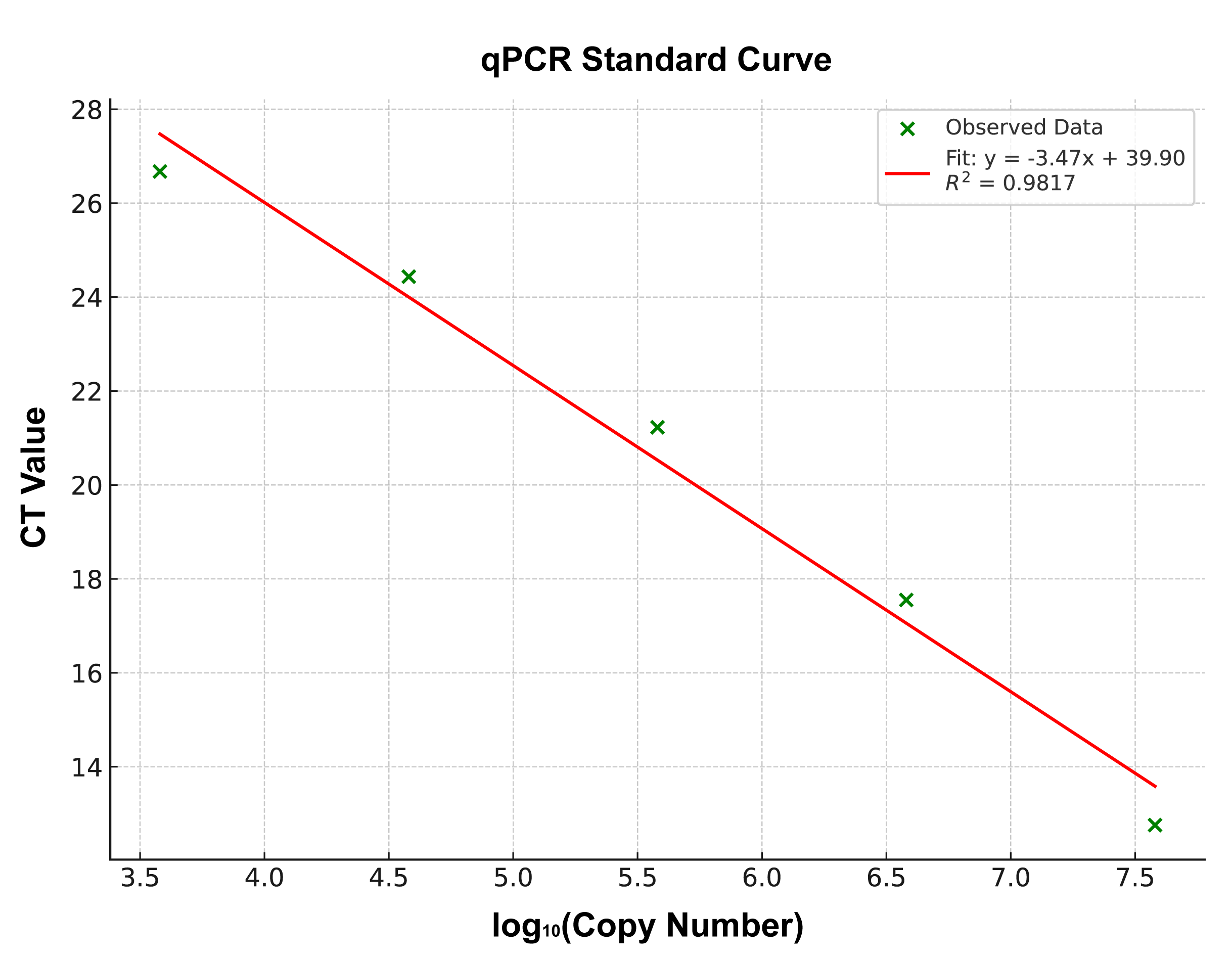

Supplement: Supplementary file 5 — Supplementary Material 5: Fig. S5 The standard curve for quantitative real-time PCR (qPCR). [file 44297_2026_79_MOESM5_ESM.tif]
