# Supplementary material for: Leptochloa chinensis identified as a new reservoir host of southern rice black-streaked dwarf virus
Source: Crop Health. 2026 Jun 25;4(1):17. doi: 10.1007/s44297-026-00079-2 (PMC13304047; doi:10.1007/s44297-026-00079-2)
Supplement: Supplementary file 6 — Supplementary Material 6: Table S1 Primers used in this study. [file 44297_2026_79_MOESM6_ESM.docx]

**Supplementary Table 1 Primers used in this study.**

| **Primer** | **Sequence (5' to 3')** | **Purpose** |
| --- | --- | --- |
| ITS4 | TCCTCCGCTTATTGATATGC | Identification of weed species |
| ITS5 | GGAAGTAAAAGTCGTAACAAGG |  |
| SRBs1-1F | AAGTTTTTTTCGCCGACCTACGTATCTC | SRBSDV segment 1 full-length verification |
| SRBs1-1R | CTTCATCATTAGTTAGCTCTCTGGA |  |
| SRBs1-2F | ACTGACCTTGGTAACATTGT |  |
| SRBs1-2R | CACTCCATTCTTGATAACCTC |  |
| SRBs1-3F | GTTGTTATCCATGCCGCTAAT |  |
| SRBs1-3R | CTTTCTTCGAGAGATTCTGTTC |  |
| SRBs1-4F | CGAACAACACATGTTCCAAT |  |
| SRBs1-4R | GACGACAGCTGTTTTCGCCATGGACAC |  |
| qSRBs10-1F | CGACCAACAATCACACTTC | SRBSDV quantitative PCR |
| qSRBs10-1R | GTTGAGTAATGTTCGGTAGAG |  |

Abbreviations: SRBSDV, Southern rice black-streaked dwarf virus.
